# Assessing the Relationship between Indexed Epicardial Adipose Tissue Thickness, Oxidative Stress in Adipocytes, and Coronary Artery Disease Complexity in Open-Heart Surgery Patients

**DOI:** 10.3390/medicina60010177

**Published:** 2024-01-19

**Authors:** Laurentiu Braescu, Adrian Sturza, Oana Maria Aburel, Raluca Sosdean, Danina Muntean, Constantin Tudor Luca, Daniel Miron Brie, Horea Feier, Simina Crisan, Cristian Mornos

**Affiliations:** 1 Department VI Cardiology—Cardiovascular Surgery Clinic, Institute for Cardiovascular Diseases of Timișoara, “Victor Babeș” University of Medicine and Pharmacy, E. Murgu Sq. No. 2, 300041 Timișoara, Romania; braescu.laurentiu@umft.ro; 2 Doctoral School Medicine-Pharmacy, “Victor Babeș” University of Medicine and Pharmacy, E. Murgu Sq. No. 2, 300041 Timișoara, Romania; 3 Department III Functional Sciences—Pathophysiology, “Victor Babeș” University of Medicine and Pharmacy, E. Murgu Sq. No. 2, 300041 Timișoara, Romania; sturza.adrian@umft.ro (A.S.); oanaduicu@umft.ro (O.M.A.); daninamuntean@umft.ro (D.M.); 4 Center for Translational Research and Systems Medicine, “Victor Babeș” University of Medicine and Pharmacy, E. Murgu Sq. No. 2, 300041 Timișoara, Romania; 5 Department VI Cardiology—Cardiology Clinic, Institute for Cardiovascular Diseases of Timișoara, “Victor Babeș” University of Medicine and Pharmacy, E. Murgu Sq. No. 2, 300041 Timișoara, Romania; sosdean.raluca@umft.ro (R.S.); ctluca@cardiologie.ro (C.T.L.); brie_daniel@yahoo.com (D.M.B.); urseanusimina@yahoo.com (S.C.); mornos.cristian@umft.ro (C.M.); 6 Research Center of the Institute of Cardiovascular Diseases Timișoara, “Victor Babeș” University of Medicine and Pharmacy, E. Murgu Sq. No. 2, 300041 Timișoara, Romania

**Keywords:** epicardial fat, oxidative stress, coronary artery disease

## Abstract

*Background and Objectives*: This cross-sectional study conducted at the Timișoara Institute of Cardiovascular Diseases, Romania, and the Centre for Translational Research and Systems Medicine from “Victor Babeș” University of Medicine and Pharmacy of Timișoara, Romania, investigated the relationship between indexed epicardial adipose tissue thickness (EATTi) and oxidative stress in epicardial adipose tissue (EAT) adipocytes in the context of coronary artery disease (CAD) among open-heart surgery patients. The objective was to elucidate the contribution of EATTi as an additional marker for complexity prediction in patients with CAD, potentially influencing clinical decision-making in surgical settings. *Materials and Methods*: The study included 25 patients undergoing cardiac surgery, with a mean age of 65.16 years and a body mass index of 27.61 kg/m^2^. Oxidative stress in EAT was assessed using the ferrous iron xylenol orange oxidation spectrophotometric assay. The patients were divided into three groups: those with valvular heart disease without CAD, patients with CAD without diabetes mellitus (DM), and patients with both CAD and DM. The CAD complexity was evaluated using the SYNTAX score. *Results*: The EATTi showed statistically significant elevations in the patients with both CAD and DM (mean 5.27 ± 0.67 mm/m^2^) compared to the CAD without DM group (mean 3.78 ± 1.05 mm/m^2^, *p* = 0.024) and the valvular disease without CAD group (mean 2.67 ± 0.83 mm/m^2^, *p* = 0.001). Patients with SYNTAX scores over 32 had significantly higher EATTi (5.27 ± 0.66 mm/m^2^) compared to those with lower scores. An EATTi greater than 4.15 mm/m^2^ predicted more complex CAD (SYNTAX score >22) with 80% sensitivity and 86% specificity. The intra- and interobserver reproducibility for the EATTi measurement were excellent (intra-class correlation coefficient 0.911, inter-class correlation coefficient 0.895). *Conclusions*: EATTi is significantly associated with CAD complexity in patients undergoing open-heart surgery. It serves as a reliable indicator of more intricate CAD forms, as reflected by higher SYNTAX scores. These findings highlight the clinical relevance of EATTi in pre-operative assessment, suggesting its potential utility as a prognostic marker in cardiac surgical patients.

## 1. Introduction

Epicardial adipose tissue (EAT), located between the myocardium and the epicardium and supplied by the coronary arteries, is increasingly recognized for its role in the development and progression of coronary artery disease (CAD) [1]. This association has spurred interest in non-invasive imaging techniques, particularly echocardiography, to assess EAT as a marker for cardiovascular disease risk.

Standard 2D echocardiography can be considered as a reliable method for visualizing and measuring EAT thickness (EATT). This technique is particularly effective in assessing the free wall of the right ventricle, where the EAT layer is thickest [2], and is best performed from parasternal long- and short-axis views for optimal accuracy [1,3,4].

An EATT greater than 4 mm is an established independent predictor of major adverse cardiovascular events [5]. Studies have shown that elevated an EATT of 7 mm in men and 6.5 mm in women correlates with increased left ventricular mass, diastolic dysfunction, and greater carotid stiffness and intima-media thickness [6]. Additionally, acute myocardial infarction patients with an EATT over 4 mm tend to have larger infarct sizes and poorer left ventricular systolic function [1,5]. EAT has also been gaining interest regarding oxidative stress [1,7,8]. The EATT was related with endothelial dysfunction, and it was concluded that EAT may predict the early reversible stages of atherosclerosis [3]. Recently, it has been proposed that patients with severe coronary atherosclerosis, glucose and insulin metabolic disorder, and serum adiponectin reduction present a higher concentration of reactive oxygen species (ROS) [9].

In addition to the established cardiovascular implications, EATT is increasingly implicated in the metabolic dysregulation seen in diabetes. Diabetic individuals typically exhibit increased EATT, which secretes pro-inflammatory cytokines exacerbating insulin resistance and atherosclerosis [10]. This heightened inflammatory and oxidative state in EAT among diabetics underscores its potential as a critical marker for cardiovascular risk in this population.

While echocardiography is advantageous for its accessibility and reproducibility, it is limited by its operator-dependent nature. The current guidelines advocate for the indexation of echocardiographic measures using body surface area (BSA), enhancing the prognostic performance of these measurements [11].

Our study focuses on investigating the relationship between indexed epicardial adipose tissue thickness to BSA (EATTi) and oxidative stress in EAT adipocytes in the context of CAD complexity among open-heart surgery patients. This investigation seeks to elucidate the contribution of EAT and associated oxidative stress to CAD, potentially influencing clinical decision-making and risk stratification in surgical settings.

## 2. Materials and Methods

### 2.1. Study Design and Participants

Conducted at the Institute of Cardiovascular Diseases in Timișoara, Romania, and the Centre for Translational Research and Systems Medicine from “Victor Babeș” University of Medicine and Pharmacy of Timișoara, Romania, this study was designed as a cross-sectional analysis focusing on the relationship between epicardial and periaortic fat thickness and CAD outcomes in patients undergoing open-heart surgery. Prior to surgery, informed consent was obtained from all the participants, in line with the ethical standards of the Declaration of Helsinki as established by the World Medical Association.

Patient selection was guided by a set of predefined criteria, aiming to create a diverse and representative sample, totaling 25 patients who underwent open-heart surgery and from whom EAT samples were taken to perform various measurements. The study protocol and all the associated procedures were rigorously reviewed and approved by the Commission for Research Ethics of the university (approval number 09 from 22 March 2021). To ensure the confidentiality and privacy of patient data, all the identifying information was removed, and the data were anonymized prior to analysis. The research adhered to the EU Good Clinical Practice Directives (2005/28/EC) and ICH guidelines and received clearance from the Local Commission of Ethics for Scientific Research (number 371 from 20 January 2021).

### 2.2. Inclusion and Exclusion Criteria

The study’s inclusion criteria encompassed patients referred for cardiac surgery, which included individuals diagnosed with coronary artery disease and those presenting with valvular heart conditions but without CAD. Adult patients aged 18 years or older were eligible for participation. Additionally, all the participants were required to provide informed consent, agreeing to the use of their biological samples and medical data for the purposes of the study.

On the other hand, the exclusion criteria were designed to ensure the accuracy and relevance of the study’s findings. Patients with poor echocardiographic windows, which could compromise the precision of the cardiac assessments, were excluded. Individuals with any form of neoplasia were also excluded to prevent the confounding effects of cancer or its treatments on cardiac and adipose tissue health. Moreover, patients with chronic inflammatory or autoimmune diseases were not included due to the potential independent impact of these conditions on cardiovascular health and adipose tissue. Active infections or chronic hepatic diseases were additional exclusion criteria, owing to their potential systemic effects that could influence the study results. Lastly, patients with congenital heart disease were excluded to maintain the study’s focus on acquired cardiac conditions rather than congenital anomalies.

### 2.3. Definitions and Variables

#### 2.3.1. Echocardiography

Echocardiographic evaluations were performed one day prior to surgery using a Vivid E95 ultrasonographic system from General Electric. These assessments included conventional echocardiography, tissue Doppler imaging, and speckle tracking imaging. Two experienced sonographers, blinded to the patient’s clinical presentation and clinical workup results, performed echocardiography in a bedside manner. The key measurements included left ventricular (LV) end-diastolic and end-systolic volumes and diameters, LV ejection fraction (LVEF), and left atrial maximum volume and area, all conducted according to established guidelines [11].

The epicardial adipose tissue thickness was measured perpendicularly to the free wall of the right ventricle during three cardiac cycles at end systole, utilizing a parasternal long-axis view [2]. This approach ensured the accurate measurement of EAT at its thickest point in each cycle, with the mean EATT calculated as an average across the cycles. The indexed EATT was calculated as the ratio between the EATT and body surface area. The transmitral flow patterns and maximal velocities of the E wave were recorded from an apical four-chamber view using a 5 mm pulsed-sample Doppler volume and were averaged over three consecutive cardiac cycles [11,12].

The mitral annulus motion parameters, including peak systolic velocity (s’) and early diastolic velocity (e’), were recorded using pulsed tissue Doppler imaging in the apical four-chamber view, with measurements taken at the lateral and septal corners of the mitral annulus to calculate the average E/e’ ratio [11,12]. The LV global longitudinal strain (GLS) was determined from apical four-, three-, and two-chamber views using a 17-segment model of the LV [13], with the average GLS computed from the remaining suitable apical views after excluding segments that failed to track.

#### 2.3.2. The SYNTAX I and II Scores

In our study, the SYNTAX score was used as a metric for assessing the complexity of coronary artery lesions. This score was derived by summing up the individual scores for each coronary lesion with ≥50% luminal obstruction in vessels ≥1.5 mm in diameter, thereby aiding cardiologists and surgeons in evaluating the intricacy of coronary pathologies [14]. A higher SYNTAX score generally indicates more complex coronary lesions and is often associated with a poorer prognosis in patients undergoing revascularization procedures.

To ensure the objectivity of the SYNTAX score, two interventional cardiologists, blinded to the patients’ echocardiographic and clinical data, independently performed the angiographic scoring. The scores assigned by these observers were averaged to determine each patient’s final SYNTAX score, with any significant discrepancies resolved through a re-scoring process. The scores were then categorized into low (<23), intermediate (23–32), and high (>32) tertiles using specialized software for calculation [15]. This categorization facilitated a more detailed analysis of the correlation between the complexity of coronary artery lesions and other cardiac parameters in the study.

The SYNTAX score is a comprehensive tool designed to evaluate the complexity of coronary artery lesions, considering factors such as bifurcations, chronic total occlusions, thrombus, calcification, and small diffuse disease, with its range extending from 0 to over 60 in cases of highly complex coronary anatomy. This scoring system serves as an angiographic tool aiding cardiologists, interventionists, and surgeons in grading the intricacy of coronary artery lesions. A higher SYNTAX score signifies increased complexity and a poorer prognosis, particularly in patients undergoing contemporary revascularization, notably with percutaneous coronary intervention (PCI) [16]. Enhancing its predictive capabilities, the SYNTAX score II integrates both anatomic and clinical prognostic variables to provide accurate mortality predictions, thereby guiding the decision-making process between PCI and coronary artery bypass grafting (CABG) for individuals with multivessel coronary disease. Additionally, the residual SYNTAX score (rSS) has been developed to quantify the adverse effects of incomplete coronary revascularization, obtained post-revascularization. In clinical application, a SYNTAX score of 22 or lower suggests that PCI might be a viable alternative to CABG for patients with low anatomical complexity. Conversely, for those with a SYNTAX score exceeding 22, CABG is typically recommended due to its superior clinical outcomes compared to PCI.

#### 2.3.3. Adipose Tissue Assessment

In the course of our study, epicardial and periaortic (perivascular) fat specimens were systematically collected from patients undergoing open-heart surgery, specifically after the initiation of cardiopulmonary bypass. The samples of EAT, each weighing between 0.5 to 1 g, were meticulously harvested during the surgical procedure. These explants constituted the primary material for our subsequent analysis. The epicardial and periaortic adipose tissue explants were used for culture to analyze their biochemical and inflammatory characteristics. The adipocytes were not isolated individually; instead, whole tissue explants were cultured to maintain the complex interplay of cells within the adipose tissue environment, essential for understanding the pathophysiology of CAD and its interplay with EAT. The explants were placed in a sterile culture medium designed to support adipose tissue and incubated at 37 °C in a 5% CO_2_ atmosphere. The medium was refreshed every 48 h, and the supernatants were collected at specific time points for subsequent analysis of the secreted factors.

#### 2.3.4. Oxidative Stress Assessment

Oxidative stress within the EAT samples was quantified by means of the ferrous iron xylenol orange oxidation (FOX) assay. This method uses a PeroxiDetect Kit (Sigma Aldrich, Burlington, MA, USA) to assess the H_2_O_2_ levels according to a previously described technique [17,18]. The principle of this assay is that H_2_O_2_ oxidizes ferrous iron (Fe^2+^) to ferric iron (Fe^3+^) at acidic pH. The latter forms a colored adduct with xylenol orange, which is measured spectrophotometrically at 560 nm. The results were expressed in nanomoles of H_2_O_2_ per milligram of tissue per hour, using a standard curve for precise quantification.

#### 2.3.5. Body Surface Area

In this study, the body surface area (BSA) of the participants was calculated using the Mosteller formula, one of the most widely accepted methods for BSA determination in medical practice [19]. This formula is defined as the square root of the product of the patient’s weight in kilograms and height in centimeters divided by 3600. Specifically, BSA (m^2^) = √([Height(cm) × Weight(kg)]/3600). This method was chosen for its simplicity and proven clinical relevance, providing a standardized measure to index various physiological and anatomical parameters, including cardiac output and drug dosing.

#### 2.3.6. Comorbidities and Medication

Diabetes was defined as a history of diabetes mellitus type 2 diagnosed by a healthcare professional, based on criteria from the American Diabetes Association, which includes fasting plasma glucose levels of 126 mg/dL or higher, HbA1c of 6.5% or higher, or being on antidiabetic medications [20]. Smoking status was categorized as current smoker, former smoker, or never a smoker. Current smokers were defined as individuals who have smoked at least 100 cigarettes in their lifetime and currently smoke cigarettes. Former smokers were those who have smoked at least 100 cigarettes in their lifetime but had quit at the time of the study. Hypertension was defined according to the American Heart Association guidelines as systolic blood pressure readings of 130 mmHg or higher, or diastolic blood pressure readings of 80 mmHg or higher on two separate occasions or currently taking antihypertensive medication [21].

In this study, among 25 patients, various medications were administered with the following distribution: Aspirin was taken by 14 patients, constituting 56.0% of the total. Other antiplatelets were used by 11 patients, making up 44.0%. Beta-blockers were prescribed to 20 patients, accounting for 80.0% of the sample. Anticoagulants were given to 3 patients (12.0%), while statins were administered to 17 patients (68.0%). Ezetimibe was used by 4 patients, corresponding to 16.0%. Nitrates were prescribed to 7 patients (28.0%), and amlodipine to 9 patients (36.0%). ACE inhibitors and ARBs were each used by 9 and 7 patients, making up 36.0% and 28.0%, respectively. A significant 92.0% (23 patients) received diuretics, and furosemide was given to 16 patients (64.0%). Spironolactone was administered to 14 patients (56.0%), and indapamide to 5 patients (20.0%). Only one patient received digoxin (4.0%). Insulin was prescribed to 3 patients (12.0%), and ADO to 5 patients (20.0%).

### 2.4. Statistics

To conduct these statistical analyses, we utilized the SPSS software package, version 26 (SPSS Inc., Chicago, IL, USA). In our study, numerical variables were represented as mean ± standard deviation, and comparisons between groups were conducted using Student’s *t*-tests or analysis of variance (ANOVA), depending on the data distribution and study design requirements. The Shapiro–Wilk test was used for assessing the normality of data, being particularly suitable for smaller sample sizes, while a multiple linear regression was performed using the normally distributed variables. Categorical variables were presented as absolute numbers and percentages, and their comparisons were made using the χ2 (Chi-square) tests. A random subset of 15 patients was selected to evaluate the reproducibility of the EATT measurements, and it was assessed using intra-class and inter-class correlation coefficient. Throughout our analysis, a *p*-value of less than 0.05 was considered to indicate statistical significance.

## 3. Results

The study comprised 25 participants referred for open-heart surgery, with a mean age of 65 years and a mean body mass index (BMI) over 27 kg/m^2^. Regarding the CAD complexity, as measured by the SYNTAX score, the mean value was 26.32. The mean systolic blood pressure SBP was 140.8 mmHg, while the mean diastolic blood pressure DBP was 84.68 mmH. In terms of comorbidities and risk factors, 28% of the participants were diagnosed with stage 3 hypertension. Hereditary factors were present in 16% of participants. Diabetes and smoking were each noted in 28% of participants, while a significant 84% had dyslipidemia. Additionally, 56% of the participants had confirmed coronary artery disease, as presented in Table 1.

Table 2 of the study delineates a comparative analysis of the background data of 25 participants, categorized into Non-CAD (*n* = 11), CAD-DM (*n* = 10), and CAD+DM (*n* = 4) groups. The BMI across the groups does not show a statistically significant variation. The SYNTAX I score in the CAD+DM group had a notably higher mean score compared to the CAD-DM group, the difference being statistically significant. The SYNTAX II score followed a similar trend, with the CAD+DM group showing a higher mean value, although the difference was not statistically significant. The blood pressure readings, both systolic (SBP) and diastolic (DBP), are higher on average in the CAD-DM and CAD+DM groups compared to Non-CAD, but these differences were not statistically significant.

The analysis of the lipid profiles showed that the mean low-density lipoprotein (LDL) cholesterol level was 112.40 mg/dL. The mean level of triglycerides was 159.20 mg/dL. The total cholesterol levels averaged at 181.88 mg/dL. The hemoglobin levels were found to be within normal limits, with a mean value of 13.72 g/dL, while the leukocyte count was 7433.64 cells/mm^3^. The renal function, as assessed by the mean creatinine level and estimated glomerular filtration rate (eGFR), was within normal limits. The mean creatinine was 0.99 mg/dL, while the eGFR was 86.97 mL/min, suggesting overall adequate renal function in the cohort.

The liver function tests were within normal ranges, with mean AST levels of 22.28 U/L, while ALT was 27.56 U/L. Electrolytes, including sodium and potassium, were within normal limits, with mean levels of 141.28 mmol/L for sodium and 4.19 mmol/L for potassium. The high-sensitivity C-reactive protein (hs-CRP) levels averaged at 1.36 mg/L. Finally, markers of oxidative stress in the adipocytes, specifically in the EAT and perivascular regions, were measured. The mean ROS levels in the EAT were 18.92 nmol H_2_O_2_/g tisssue/h, and the perivascular ROS levels averaged at 19.20 nmol H_2_O_2_/g tisssue/h, suggesting a heightened state of oxidative stress in these tissues, as presented in Table 3.

**Figure 1:**
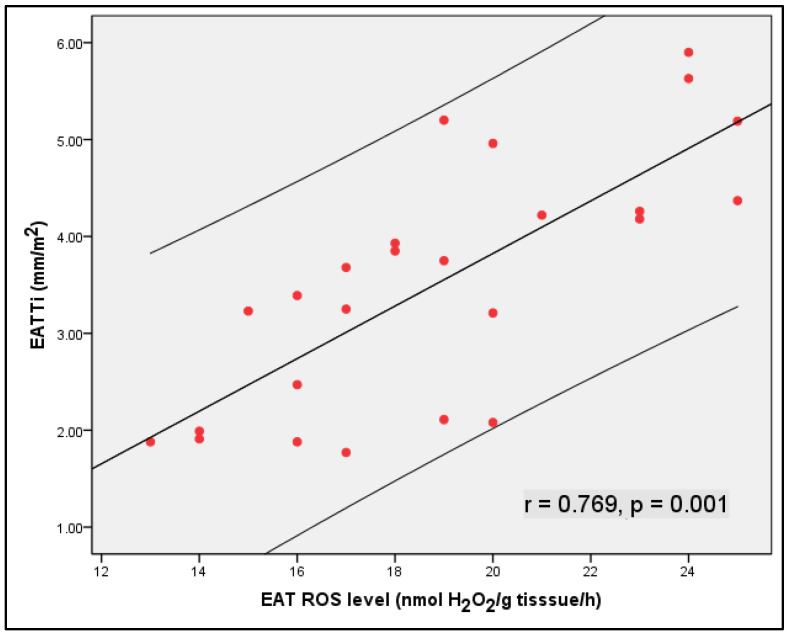
Scatter plot of the relationship between epicardial adipose tissue thickness indexed to the body surface area (EATTi) and epicardial adipose tissue reactive oxygen species (ROS) levels in patients referred for heart-open surgery.

The epicardial adipose tissue measurement, according to the Iacobelli method and the indexed epicardial adipose tissue thickness (Figure 1), both showed a highly significant correlation with the EAT ROS levels (*p*-value < 0.001), indicating that greater epicardial adipose tissue thickness is associated with increased oxidative stress in epicardial adipose tissue (Table 3).

Of the conventional echocardiographic parameters, the left atrial volume index (LAVI) also demonstrated a significant correlation with EAT ROS (*p*-value = 0.002), suggesting a relationship between left atrial enlargement and increased oxidative stress in epicardial adipose tissue. A similar significant correlation was observed with the left atrial (LA) diameter and EAT ROS (*p*-value = 0.014). The left ventricular (LV) mass showed a significant correlation with EAT ROS (*p*-value = 0.043), implying a relationship between increased LV mass and higher oxidative stress in epicardial adipose tissue. The end-diastolic dimension of the left ventricle (EDDLV) also correlated significantly with EAT ROS (*p*-value = 0.038). The pulmonary artery systolic pressure (PASP) levels were significantly correlated with EAT ROS (*p*-value of 0.010), indicating that higher PASP levels are associated with increased oxidative stress in epicardial adipose tissue (Table 3).

Perivascular fat ROS levels correlated well with EATTi and EATT (*r* = 0.665 and 0.636, respectively, both *p*-value = 0.001), EDDLV (*r* = 0.458, *p*-value = 0.021), and LAVI (*r* = 0.431, *p*-value = 0.029), as shown in Table 4.

A multiple linear regression model, which included EATTi and the statistically significant conventional echocardiographic parameters (EDDLV, LAVI, LV mass, and PASP) as determinants of the intensity of ROS epicardial fat tissue production, was also constructed (Table 5). A key finding was the positive β coefficient of 1.786 for EATTi, which was statistically significant (*p*-value = 0.001) (as presented in Table 4), indicating that increased EATT was associated with higher oxidative stress in the epicardial tissue adipocytes.

The multiple linear regression model revealed a significant association between EATTi and oxidative stress in epicardial tissue adipocytes, with a cutoff value of 22 (*p*-value = 0.001). The model exhibited a sensitivity of 74.9% and specificity of 77.1%, indicating moderate accuracy in identifying cases with increased oxidative stress due to EATTi. With an area under the curve (AUC) of 0.686, the model demonstrates a reasonable ability to discriminate between patients with and without increased oxidative stress, suggesting its potential utility in a clinical setting.

Conversely, the LAVI showed a β coefficient of 0.335. Although not statistically significant (*p*-value = 0.131), this suggests that LAVI may not be a reliable determinant of EAT ROS levels in this cohort. End-diastolic dimension of the left ventricle, with a β coefficient of 0.396, also did not reach statistical significance (*p*-value = 0.073), similarly to the LV mass. Pulmonary artery systolic pressure (PASP) had a β coefficient of 0.235, but this relationship was not statistically significant (*p*-value = 0.122).

**Figure 2:**
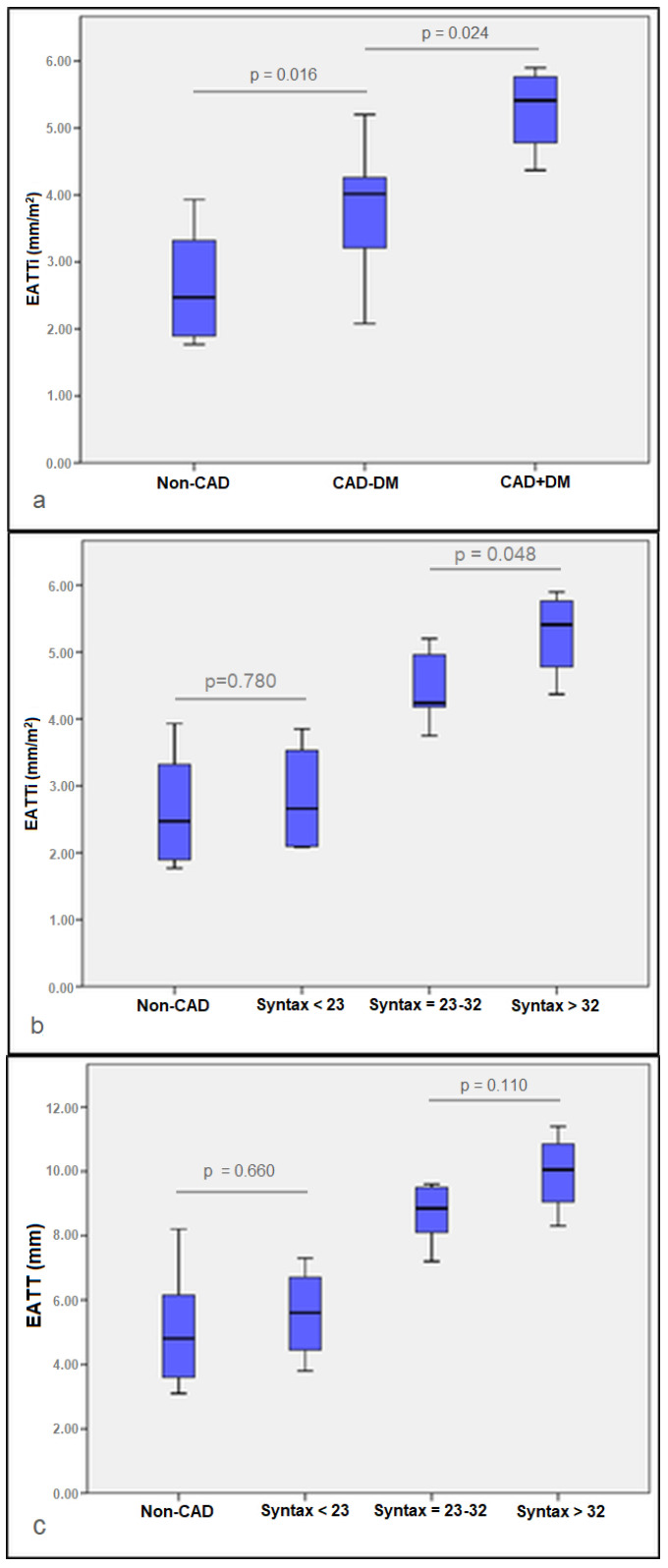
Boxplot analysis of epicardial fat thickness: (**a**) subgroup analysis of patients referred for cardiac surgery: valvular heart disease but devoid of coronary artery disease (Non-CAD), a second group with CAD in the absence of diabetes mellitus (CAD-DM group), and a third group with both CAD and DM (CAD+DM group); (**b**) EATTi vs. SYNTAX score groups; (**c**) EATT vs. SYNTAX score groups. Data presented as median (IQR).

In the comparative analysis of the echocardiographic parameters across the study groups, it was observed that the EATTi exhibited statistically significant elevations in the CAD+DM group in comparison to both the CAD-DM group (*p* = 0.024) and the Non-CAD group (*p* = 0.001). Additionally, a notable increase in EATTi was discernible in the CAD-DM group relative to the Non-CAD group (*p* = 0.016), as presented in Figure 2a.

Furthermore, when categorizing the CAD patients based on their SYNTAX scores, a notable finding was the heightened EATTi levels in patients with SYNTAX scores exceeding 32, contrasted against those with scores ranging from 23 to 32 (*p* = 0.048) and scores below 23 (*p* = 0.005). Interestingly, the EATTi levels in patients with SYNTAX scores under 23 were found to be similar to those in patients without CAD (*p* = 0.78) (Figure 2b).

In the context of the EATT measurements, no statistical significance was observed between patients with SYNTAX scores over 32 and those with scores between 23 and 32. However, both groups exhibited significantly higher EATT levels compared to the cohort with SYNTAX scores below 23. Additionally, the EATT measurements in patients with SYNTAX scores under 23 were found to be comparable to those in patients without CAD, as seen in Figure 2c.

**Figure 3:**
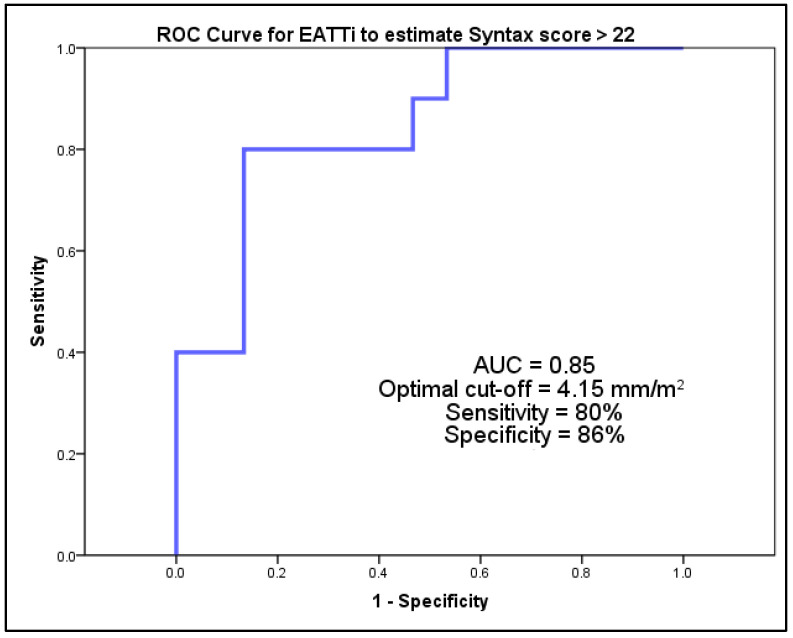
Receiver operating characteristic (ROC) curves for EATTi to predict coronary artery disease with a SYNTAX score > 22 in patients referred for open-heart surgery.

In this cohort of patients referred for cardiac surgical intervention, the investigation revealed that an EATTi exceeding 4.15 mm/m^2^ serves as a reliable marker for the identification of individuals with more intricate forms of CAD, as indicated by a SYNTAX score surpassing 22. This was substantiated by the observed sensitivity of 80% and a specificity of 86% for this EATTi threshold. The findings are illustrated in Figure 3. The intra-observer reproducibility calculated from a subset of 15 random patients was excellent, with an intra-class correlation coefficient value of 0.911 (95%CI = 0.883–0.939) and an inter-class correlation coefficient value of 0.895 (95%CI = 0.872–0.918).

## 4. Discussion

### 4.1. Study Implications and Literature Analysis

The current study is the first to explore the connection between EATTi, oxidative stress parameters, and CAD complexity in the patient cohort referred for cardiac surgery. The findings indicate a significant correlation between EATTi and oxidative stress within the epicardial adipose tissue, establishing EATTi as a potent predictor of complex CAD requiring revascularization therapy. Thus, our investigation offers a new perspective on the interplay between adipose tissue thickness and cardiac pathology, paving the way for different approaches in cardiovascular disease management and treatment strategies [1,22,23,24,25].

EAT, a metabolically active visceral fat deposit situated in the epicardial space and directly in contact with the heart, has gained considerable attention in recent medical research. This paracrine organ, nestled between the visceral pericardium and the outer myocardial surface, exerts significant influence on both local and systemic inflammatory and atherogenic processes through adipokine production and secretion [1,6,26,27,28]. EAT’s direct linkage to CVD is increasingly recognized as more impactful than general obesity [2,9,29,30]. The disruption of the balance between EAT’s cardioprotective and deleterious effects contributes systemically to the development and progression of CAD. Excessive adiposity in EAT has been shown to release inflammatory cytokines, augmenting endothelial dysfunction, increasing oxidative stress, and, consequently, accelerating coronary atherosclerosis [8,9,22]. A growing body of evidence suggests that excessive epicardial adiposity is an independent risk factor for CVD. Increased EATT symbolizes a clinical indicator of excessive visceral fat accumulation [4,8,11,16]. Despite certain limitations, two-dimensional echocardiography remains the preferred method for EAT assessment in daily clinical practice, favored for its low cost, accessibility, and reproducibility [2,6,22].

Several studies have proposed different thresholds for EATT, underscoring its substantial association with atherogenic risk factors. For instance, Iacobellis identified an average EATT of 7 mm in men and 6.5 mm in women as standard clinical benchmarks [2,6]. Islas et al. found that patients with acute myocardial infarction and EAT thickness over 4 mm exhibited deteriorated left ventricular systolic function and larger infarct sizes [5]. In patients with essential hypertension, an EATT greater than 7 mm was linked to an elevated left ventricular mass index and diastolic dysfunction [4]. Furthermore, an EATT exceeding 4 mm has been reported to predict major adverse cardiovascular events with considerable accuracy over a 5-year follow-up [5]. Jehn et al.’s findings indicate that an EATT of 5.5 mm could be a valuable echocardiographic marker for detecting obstructive CAD in patients presenting with acute chest pain [23].

In the context of EATT and its correlation with cardiovascular risks, it is essential to note the varying technical methodologies employed across the referenced studies. The studies by Iacobellis and Jehn et al. [2,6] utilized end-systolic measurements, capturing the EATT at the heart’s most contracted state. In contrast, the work by Islas et al. [5] took measurements at end-diastole, when the heart is in its most relaxed form. This distinction is not just a technicality but a fundamental aspect that can influence the interpretation and applicability of EATT as a clinical marker. The different phases of the cardiac cycle can significantly alter the thickness of the epicardial fat layer, thereby affecting the cutoff values determined for risk stratification. It underscores the necessity for a standardized approach in measuring EATT to ensure consistency and reliability in its use as a prognostic tool in various cardiac conditions. 

The association between epicardial fat volume and anthropometric measurements has been established, highlighting the importance of adjusting the amount of EAT for body size. This approach is vital, as demonstrated by a robust correlation between EAT volume and body size metrics [25]. Consistent with the current echocardiographic guidelines, it is recommended to index cardiac measures to BSA, regardless of the specific echocardiographic parameter in focus [11,31]. Indexing to BSA has been shown to enhance prognostic accuracy compared to unindexed measures, with no other body size indexation metric offering significant improvement in prognostic performance [31]. This study extends the examination of indexation metrics with varying exponents of height and weight to EATT measurements, thereby affirming the utility of this approach in evaluating EATT.

Moreover, the relationship between EATT indexed to BSA and the complexity of CAD, as quantified by the SYNTAX score, was examined. The SYNTAX score, derived from the aggregate of individual scores for each lesion with ≥50% luminal obstruction in vessels ≥1.5 mm, serves as an angiographic tool aiding cardiologists, interventionists, and surgeons in assessing the complexity of coronary artery lesions [14]. Erkan et al. previously demonstrated a significant correlation between the mean EATT and critical atheromatosis, as indicated by SYNTAX scores [15]. Furthermore, Mahmoud et al. reported a mean EATTi value of 2.6 ± 1.3 mm/m^2^ in patients with coronary microvascular dysfunction, underscoring EATTi’s independent association with this condition and its ability to differentiate patients with and without microvascular alterations [29].

Our findings reveal that EATTi exhibits high accuracy in predicting more complex vascular disease, particularly in cases where the SYNTAX score exceeds 22. Specifically, an EATTi greater than 4.15 mm/m^2^ demonstrated a sensitivity of 80% and a specificity of 86% in identifying patients with intermediate to severe CAD. This indicates that EATTi is not only a reliable indicator of epicardial fat volume in relation to body size but also a significant predictor of CAD complexity, particularly in patients referred for cardiac surgical interventions.

The role of EAT in cardiovascular pathology raises a crucial question: is EAT merely a marker of visceral adiposity, signaling the presence of other cardiovascular risk factors, or does it have a causal relationship with these pathologies? [25]. The pathophysiological mechanism involving the excessive production of ROS by adipocytes, a condition termed adipocyte oxidative stress, is characterized by an overabundance of ROS and diminished antioxidant defense. This phenomenon is a recognized contributor to cardiovascular diseases, particularly those associated with metabolic disorders. Notably, EAT has been found to generate more ROS than subcutaneous adipose tissue, attributed to its higher expression of NADPH oxidase components, such as gp91phox and p47phox [30]. However, there are limited studies examining the association between the quantity of EAT and periaortic adipose tissue and the production of ROS by their adipocytes.

Valvular heart disease without concurrent CAD offers a distinct pathophysiological perspective, where the primary myocardial stress and compensatory changes, such as hypertrophy and dilation, arise from altered hemodynamics due to valvular defects, not atherosclerosis. In the context of diabetes, this condition exacerbates vascular disease through mechanisms like hyperglycemia-induced oxidative stress, the formation of advanced glycation end products, and inflammatory pathways, accelerating endothelial dysfunction and atherosclerosis [31]. The interplay between diabetes-induced molecular alterations and valvular heart disease is critical yet underexplored, highlighting the need for a deeper understanding of how diabetes-specific vascular changes impact the progression and management of valvular heart disease, distinct from those with CAD. This insight can be essential for developing targeted interventions to address the cardiovascular risks in this patient population.

Our research contributes to this area by demonstrating that the EATTi not only correlates well with EAT ROS levels but also with ROS levels in periaortic tissue. This finding underscores the potential of EATTi as a biomarker for oxidative stress in cardiac and perivascular adipose tissues. Building on these insights, our study reinforces the idea that EATT, as measured by transthoracic echocardiography and indexed to BSA, is a valuable tool for stratifying CAD risk. It suggests that indexing EATT to BSA could be particularly useful for identifying patients with increased CAD complexity. While further research on larger cohorts is necessary to consolidate these findings, our study advocates for the echocardiographic assessment of EATTi as an additional tool for predicting the complexity of CAD, bolstering the routine application of echocardiography in clinical practice. The precise quantification of EAT through these means could serve as both a critical prognostic tool and a target for CAD treatment strategies [24,32].

Echocardiographic EATTi thus emerges not only as an indicator of visceral adiposity but also as a marker for oxidative stress, implicating a more complex interplay in cardiovascular disease pathogenesis. This multifaceted role of EATTi, particularly in CAD, highlights its potential as a novel parameter in cardiovascular risk assessment and management. The implications of these findings are significant, suggesting that echocardiographic measures of EAT could pave the way for more nuanced and effective approaches in the diagnosis, risk stratification, and treatment of CAD.

### 4.2. Study Limitations

The major limitation of this study is represented by the small sample size. Our study is a single center study, and its reproduction in other centers or by multicenter studies would argue for its validity. The use of echocardiography to measure EATT has several advantages, but also has several limitations. Even if excellent interobserver and intraobserver agreement is reported, echocardiography is still an operator-dependent technique. Potential selection bias exists because the patients were selected from those referred for surgery. Currently, there are no guidelines or consensus regarding the clinical use of echocardiography for EAT measure. EAT measured on a single slice at a specific level of the heart highly correlates with measures of the total EAT burden in a study of Tran, T. et al. [33]. The presence of obstructive CAD was defined at the discretion of experienced interventional cardiologists. Additional imaging modalities, like intravascular imaging, as well as an assessment of lesion hemodynamics would have further complemented the diagnostic evaluation of these lesions; however, they were not mandatory according to the study protocol. Moreover, while the adipose tissue culture method used in our study preserves the tissue architecture and cellular interactions, we acknowledge the limitation that necrosis may occur in the inner layers of the explants over time. This potential impact on the viability and functionality of the tissues has been duly noted as a limitation in our study, and future research may consider methods for isolating and culturing individual adipocytes to complement these findings. Nevertheless, our study method provided a broad overview of oxidative stress within the epicardial adipose tissue and perivascular regions but did not differentiate between the cellular sources of ROS.

## 5. Conclusions

Our data demonstrate that in patients referred to cardiac surgery, EATTi measured by transthoracic echocardiography is related to the oxidative stress intensity in epicardial adipocytes. Indexed epicardial fat thickness seems to be a significant independent predictor of the complexity of CAD. These results support the supposition that modulation of local inflammation by epicardial fat is involved in the development of CAD. The possible input of the interplay between oxidative stress in epicardial fat and the severity of CAD requires further investigations.

## Figures and Tables

**Table 1:** Background data of the study participants.

Variables (*n* = 25)	Mean ± SD	Range
Age (years)	65.16 ± 7.71	52–80
BMI (kg/m²)	27.61 ± 4.84	18.70–37.63
SYNTAX I score	26.32 ± 8.00	8.0–38.2
SYNTAX II score	35.80 ± 14.20	18.6–61.2
SBP (mmHg)	140.80 ± 13.34	115–170
DBP (mmHg)	84.68 ± 12.06	65–105
Stage 3 hypertension, *n* (%)	7	28.0%
Metabolic syndrome, *n* (%)	16	64.0%
Heredity, *n* (%)	4	16.0%
Diabetes, *n* (%)	7	28.0%
Dyslipidemia, *n* (%)	21	84.0%
Smoking, *n* (%)	7	28.0%
CAD, *n* (%)	14	56.0%
SYNTAX I tertiles (for CAD)		
Low (<23)	4 (28.6%)	-
Intermediate (23–32)	6 (42.8%)	-
High (>32)	4 (28.6%)	-

CAD—coronary artery disease; BMI—body mass index; SD—standard deviation; *n*—number; SBP—systolic blood pressure; DBP—diastolic blood pressure.

**Table 2:** Background data of the study participants by CAD and DM groups.

Variables (*n* = 25)	Non-CAD (*n* = 11)	CAD-DM (*n* = 10)	CAD + DM (*n* = 4)	*p*-Value
Age (years)	61.82 ± 5.78	66.80 ± 8.22	70.25 ± 5.72	0.091
BMI (kg/m²)	27.59 ± 5.52	28.27 ± 4.43	26.00 ± 1.98	0.723
SYNTAX I score	–	22.80 ± 6.17	35.13 ± 2.05	0.002
SYNTAX II score	–	33.69 ± 13.18	41.10 ± 13.49	0.363
SBP (mmHg)	139.13 ± 15.62	150.34 ± 13.29	148.60 ± 13.95	0.205
DBP (mmHg)	82.44 ± 11.80	85.19 ± 13.11	84.37 ± 12.40	0.877
Stage 3 hypertension, *n* (%)	3 (27.3%)	3 (30.0%)	1 (25.0%)	0.979
Metabolic syndrome, *n* (%)	5 (45.5%)	7 (70.0%)	4 (100%)	0.132
Heredity, *n* (%)	2 (18.2%)	1 (10.0%)	1 (25.0%)	0.760
Dyslipidemia, *n* (%)	9 (81.8%)	9 (90.0%)	3 (75.0%)	0.760
Smoking, *n* (%)	2 (18.2%)	4 (40.0%)	1 (25.0%)	0.533

CAD—coronary artery disease; BMI—body mass index; SD—standard deviation; *n*—number; SBP—systolic blood pressure; DBP—diastolic blood pressure; DM—diabetes mellitus.

**Table 3:** Laboratory findings of the study participants at admission.

Variables (*n* = 25)	Mean ± SD	Range
LDL cholesterol (mg/dL)	112.40 ± 40.63	61–218
Triglycerides (mg/dL)	159.20 ± 68.69	65–391
Total cholesterol (mg/dL)	181.88 ± 59.00	100–330
Hemoglobin (g/dL)	13.72 ± 1.72	10.1–17.8
Leukocytes (cells/mm³)	7433.64 ± 1866.83	4267–11597
Creatinine (mg/dL)	0.99 ± 0.29	0.61–1.88
eFGR (ml/min)	86.97 ± 31.28	26.59–168.85
AST (U/L)	22.28 ± 7.68	10–43
ALT (U/L)	27.56 ± 19.21	10–92
Sodium (mmol/L)	141.28 ± 4.23	130–149
Potassium (mmol/L)	4.19 ± 0.34	3.70–4.90
hs-CRP (mg/dL)	1.36 ± 0.66	0.42–3.30
EAT ROS (nmol H_2_O_2_/g tisssue/h)	18.92 ± 3.55	13–25
Perivascular ROS (nmol H_2_O_2_/g tisssue/h)	19.20 ± 3.31	14–25

LDL—low-density lipoprotein; eFGR—estimated glomerular filtration rate; AST—aspartate aminotransferase; ALT—alanine aminotransferase; SD—standard deviation; *n*—number; hs-CRP—high-sensitivity C-reactive protein; EAT—epicardial adipose tissue; ROS—reactive oxygen species.

**Table 4:** Correlation analysis.

Variables (*n* = 25)	EAT ROS (ρ)	*p*-Value	Periaortic ROS (ρ)	*p*-Value
BMI	0.095	0.653	0.058	0.785
BSA	0.131	0.585	0.100	0.635
FC/min	0.023	0.913	0.182	0.383
BP systolic	0.145	0.491	0.110	0.602
BP dyastolic	0.361	0.178	0.310	0.132
LA diameter	0.486	0.014	0.311	0.130
LAVI	0.585	0.002	0.437	0.029
EDDLV	0.418	0.038	0.458	0.021
EDSLV	0.254	0.220	0.394	0.051
EF	0.080	0.703	0.193	0.354
WMSI	0.302	0.173	0.312	0.129
LV mass	0.407	0.043	0.258	0.214
TAPSE	0.303	0.142	0.306	0.137
PASP	0.508	0.010	0.259	0.212
EATT	0.746	0.001	0.636	0.001
EATTi	0.769	0.001	0.665	0.001
E/e’	0.262	0.205	0.177	0.397
s’	0.197	0.345	0.056	0.789
GLS	0.182	0.384	0.024	0.909
PCRhs	0.321	0.118	0.082	0.696
Glucose	0.459	0.021	0.227	0.276
LDL	0.017	0.937	0.141	0.502
TG	0.120	0.569	0.001	0.995
Hb	0.297	0.138	0.282	0.172
Creatinine	0.193	0.354	0.190	0.364
WBC	0.123	0.318	0.191	0.359
eGFR	0.007	0.973	0.117	0.579
ALT	0.098	0.791	0.071	0.734
ESR	0.209	0.315	0.178	0.385

BMI—body mass index; EAT_Iacobelli—epicardial adipose tissue measurement according to the Iacobelli method; EATI—epicardial adipose tissue index; PCRhs—high-sensitivity C-reactive protein; GLS—global longitudinal strain; E/e’—the ratio of early mitral inflow velocity to mitral annular early diastolic velocity (E/e’ ratio); s’—systolic velocity of mitral annulus; WMSI—wall motion score index; EF—ejection fraction; LAVI—left atrial volume index; LA diameter—left atrial diameter; LV mass—left ventricular mass; BSA—body surface area; EDDLV—end-diastolic dimension of left ventricle; EDSLV—end-systolic dimension of left ventricle; TAPSE—tricuspid annular plane systolic excursion; PASP—pulmonary artery systolic pressure; BP—blood pressure; FC/min—heart rate per minute; LDL cholesterol—low-density lipoprotein cholesterol; TG—triglycerides; Hb—hemoglobin; WBC—white blood cell count; eGFR—estimated glomerular filtration rate; ALT—alanine aminotransferase; ESR—erythrocyte sedimentation rate.

**Table 5:** A multiple linear regression model that included echocardiographic parameters as determinants of the intensity of reactive oxygen species’ epicardial fat tissue production.

Determinants of EAT ROS Levels	β Coefficient	*p*-Value
EATTi	1.786	0.001
LAVI	0.335	0.131
EDDLV	0.396	0.073
LV mass	0.344	0.231
PASP	0.235	0.122

EATTi—epicardial adipose tissue thickness index; EDDLV—end-diastolic dimension of left ventricle; LAVI—left atrial volume index; LV mass—left ventricular mass; PASP—pulmonary artery systolic pressure; ROS—reactive oxygen species.

## Data Availability

The data presented in this study are available on request from the corresponding author.
